# Radiation Dose during Digital Subtraction Angiography of the Brain—The Influence of Examination Parameters and Patient Factors on the Dose

**DOI:** 10.3390/brainsci14080799

**Published:** 2024-08-09

**Authors:** Sandra Modlińska, Jakub Kufel, Michał Janik, Łukasz Czogalik, Piotr Dudek, Marcin Rojek, Miłosz Zbroszczyk

**Affiliations:** 1 Department of Radiodiagnostics, Invasive Radiology and Nuclear Medicine, Faculty of Medical Sciences in Katowice, Medical University of Silesia in Katowice, 40-055 Katowice, Poland; sandramodlinska@gmail.com (S.M.); jakubkufel92@gmail.com (J.K.); milosz.zbroszczyk@sum.edu.pl (M.Z.); 2 Department of Radiology and Nuclear Medicine, Faculty of Medical Sciences in Katowice, Medical University of Silesia in Katowice, 40-055 Katowice, Poland; 3 Department of Biophysics, Faculty of Medical Sciences in Zabrze, Medical University of Silesia in Katowice, 40-055 Katowice, Poland; 4 Students’ Scientific Association of Computer Analysis and Artificial Intelligence, Department of Radiology and Nuclear Medicine, Faculty of Medical Sciences in Katowice, Medical University of Silesia in Katowice, 40-055 Katowice, Poland; lukczog@gmail.com (Ł.C.); piotrekd233@gmail.com (P.D.);

**Keywords:** cerebral angiography, radiation dosage, digital subtraction angiography, dose–response relationship, patient safety

## Abstract

Cerebral vascular angiography, or digital subtraction angiography (DSA), is essential for diagnosing neurological conditions but poses radiation risks. This study aims to analyze the impact of examination parameters and patient characteristics on the radiation dose received during DSA to optimize safety and minimize exposure. A retrospective analysis of 251 DSA procedures using the GE Innova IGS 630 dual-plane instrument was conducted. Data on dose area product (DAP) and air kerma (KERMA), along with patient and examination details, were collected. Statistical analyses, including Mann–Whitney, Kruskal–Wallis, and Spearman rank correlation tests, assessed the relationships between variables and radiation dose outcomes. Significant correlations were found between the sides examined (left, right, or both) and DAP (*p* < 0.0001) and KERMA (*p* < 0.0001) values, with bilateral studies showing the highest values. The post hoc Dunn tests showed that the ‘L + P’ group significantly differs from both the right group (*p* < 0.0001 and the left group (*p* < 0.0001). There is no significant difference between the ‘P’ group and the ‘L’ group (*p*-value = 0.53). These results suggest that the right and left (both) group have unique KERMA mGy values compared to the other two groups. A strong correlation (rS = 0.87) existed between DAP and KERMA. The number of projections significantly impacted radiation dose (rS = 0.61). Tube parameters (kV and mA) and skull size had low correlations with DAP and KERMA. Optimizing imaging protocols and individualizing parameters can significantly enhance patient safety and diagnostic efficacy while also reducing occupational exposure for medical staff.

## 1. Introduction

Digital subtraction angiography (DSA) of cerebral vessels is one of the most precise and effective diagnostic methods for imaging the vascular structures of the brain [1]. Its use is crucial in the diagnosis of a variety of neurological conditions, including strokes, congenital vascular pathologies such as cerebral aneurysms and arteriovenous malformations, post-traumatic defects, and brain tumors. Despite the numerous diagnostic benefits offered by DSA, the potential risks of X-ray exposure, which can adversely affect a patient’s health, cannot be overlooked [2].

The radiation dose delivered during cerebral vascular DSA is a critical aspect that requires special attention due to potential side effects, including the risk of developing secondary tumors. Various parameters of the examination, such as the duration of the procedure, the number of projections performed, the angle of projection, and the detection techniques used, have a significant impact on the total radiation dose received by the patient [3].

In addition, individual patient characteristics such as age, gender, body composition and any existing medical conditions can also have a significant impact on radiation tolerance and susceptibility. Understanding these factors and their interactions is key to optimizing imaging protocols and minimizing patient exposure to potentially adverse radiation effects [4].

In this article, we analyze the influence of various examination parameters and patient characteristics on the radiation dose received during cerebral vascular DSA, with the aim of developing strategies to ensure the most effective and safe use of this diagnostic method.

## 2. Materials and Methods

### 2.1. Study Design

A retrospective analysis was performed to investigate the relationship between examination-specific variables (number of sides examined number and type of projections made, tube parameters: kV, mA), patient characteristics (gender, presence of a coil, skull size: M8—the widest cranial width, eu left/eu right—the greatest cranial thickness near the left/right, M1—the greatest cranial length, g—the greatest cranial thickness near the point, op-the greatest cranial thickness near the point) and the conditions under which the examination itself was performed and dose area product (DAP) and air kerma (KERMA) values, for interventional radiology procedures. Skull measurements were performed on images from the analyzed study.

### 2.2. Apparatus and Protocol

All examinations were performed on a GE Innova IGS 630 dual-plane instrument (GE HealthCare Technologies Inc., Chicago, IL, USA) by a team of specialists with many years of experience in performing DSA procedures of cerebral vessels. Procedures performed in the same laboratory from 31 January 2020 to 9 April 2021 in patients with suspected or confirmed central nervous system vascular aneurysms using the Cerebral Dose Limited (Frame rate: 4 fps, duration: 5 s|Frame rate 2 fps, duration: 5 s|Frame rate: 1 fps, duration 5 s; starting parameter of X-ray tube: 75 kVa, 158.5 mA, 40 ms, filtration 0.3 mmCu.) were analyzed. DAP and KERMA values for all procedures performed were read from the dose meters immediately after the procedure and recorded in the diagnostic laboratory register. In the register of studies performed, the KERMA value was expressed in mGy, while the DAP value was expressed in µGym^2^. To convert the units from µGym^2^ to Gy-cm^2^, the DAP value was multiplied by 0.01. Both values were recorded for each X-ray tube independently, and their sums were then calculated to obtain the total DAP and KERMA value.

### 2.3. Database

The database analyzed contained 115 records relating to bilateral examinations, and 72 and 64 records relating to left- and right-sided examinations only, respectively (a total of 251 examinations). Among these studies, 192 of the 251 were related to shoulder rotation. The number of studies including the presence of embolization coils was 172. The number of studies excluding the presence of embolization coils was 124.

### 2.4. Statistical Analysis and Synthesis

Analysis of the effect of the collected parameters on the DAP and KERMA values was performed using statistical analysis methods. The analysis was performed using the Python environment (version 3.9.18) with the pandas, seaborn, matplotlib and scipy libraries. Based on the results obtained, a comprehensive assessment of the relationship between variables and the identification of significant statistical correlations was performed. *p*-values of less than 0.05 were considered statistically significant. A series of paired *t*-tests were performed to determine the association between categorical variables (sides, gender, presence of coil) and continuous variables (DAP, KERMA). As the conditions for parametric tests were not met, analyses were performed using the Mann–Whitney test (for the categorical variables gender and presence of a coil) and Kruskal–Wallis test (for the categorical variable pages as containing three variants). In addition, Spearman rank correlation analysis was performed to examine continuous variables (number of projections, DAP, KERMA, kV, mA, M8—the widest cranial width, eu left/eu right—the greatest cranial thickness near the left/right, M1—the greatest cranial length, g—the greatest cranial thickness near the point, op-the greatest cranial thickness near the point). The post hoc Dunn tests were used for further analysis.

In addition, it was checked whether there was a statistical relationship between DAP and KERMA values in the study group.

## 3. Results

**Figure 1:**
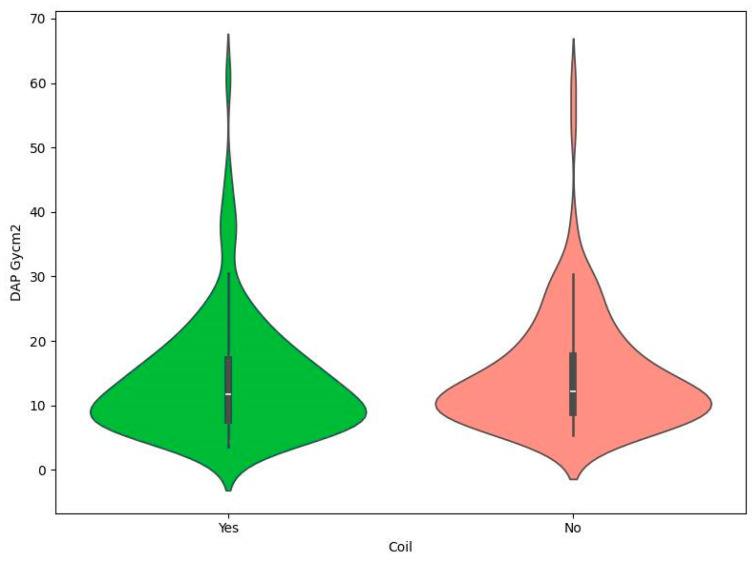
Violin plot for DAP Gycm^2^ by coil.

**Figure 2:**
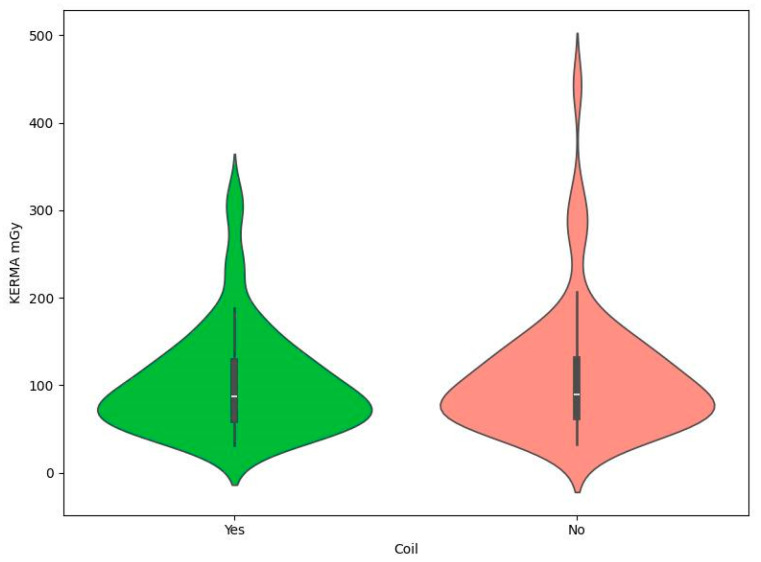
Violin plot for KERMA mGy by coil.

**Figure 3:**
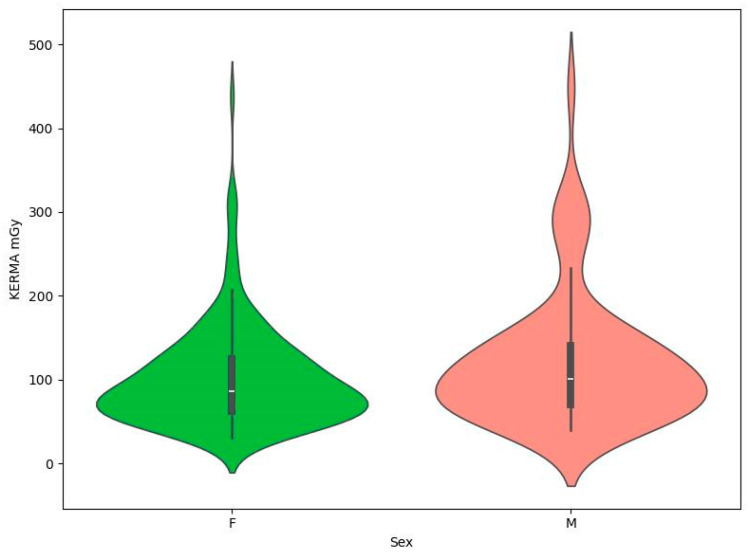
Violin plot for KERMA mGy by sex.

**Figure 4:**
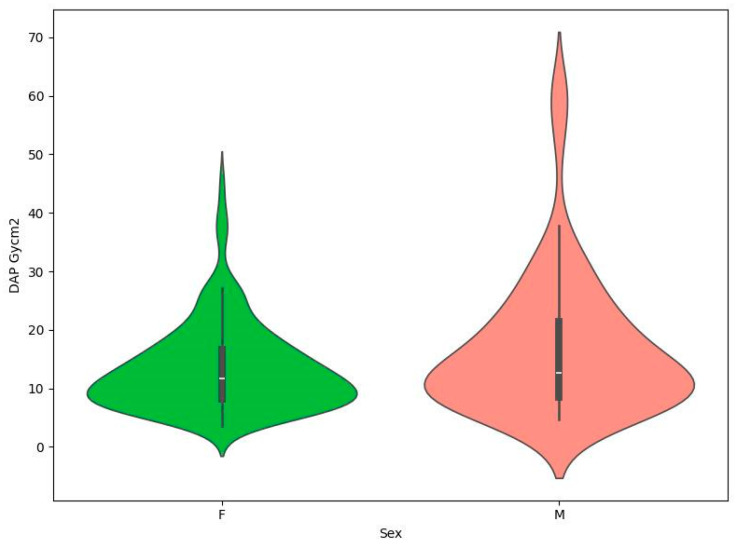
Violin plot for DAP Gycm^2^ by sex.

Patient demographics included gender: 181 female (72.11%) and 70 male (27.89%). The specific breakdown of the database in terms of demographics is shown in Table 1. This section indicates that Mann–Whitney tests showed no statistically significant relationship between gender and the presence of coils and DAP or KERMA values (Table 2). In addition, in order to visually compare DAP and KERMA values in the analyzed groups, coil graphs were made (Figure 1, Figure 2, Figure 3 and Figure 4).

### Main Findings

**Figure 5:**
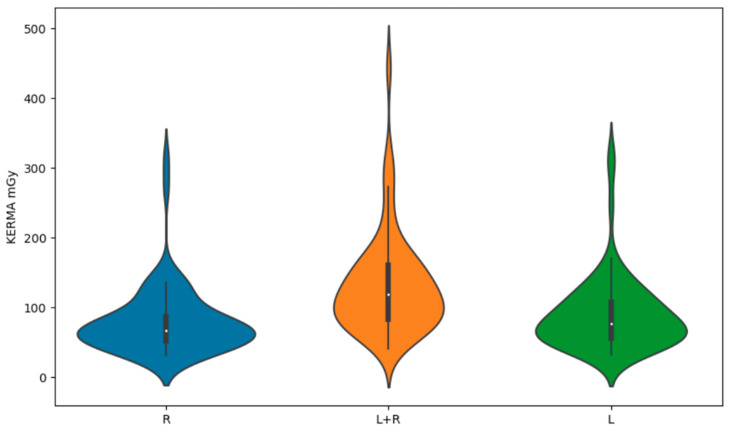
Violin plot for KERMA mGy by side.

**Figure 6:**
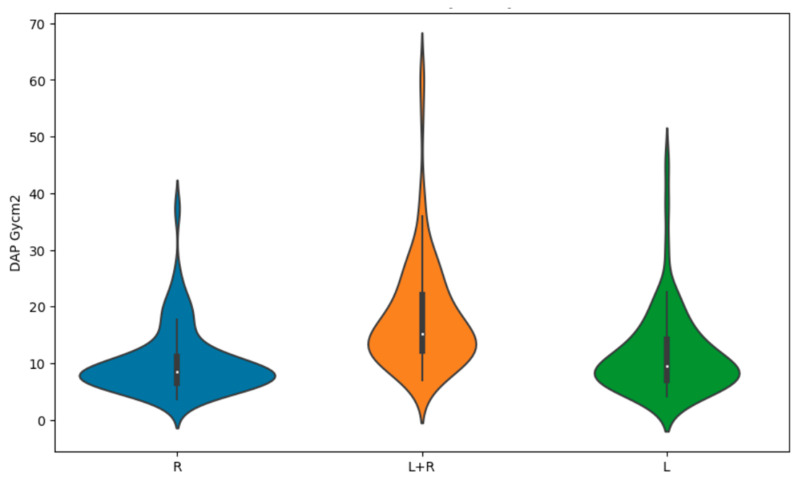
Violin plot for DAP Gycm^2^ by side.

Kruskal–Wallis tests showed statistically significant correlations between the sides considered in the study (left, right or both) and DAP (*p* < 0.0001) and KERMA (*p* < 0.0001) (Figure 5 and Figure 6). Therefore, post hoc tests were conducted to further analyze this difference. Dunn’s post hoc tests specifically showed that the ‘both’ group differs significantly from both the ‘right’ group (*p* < 0.0001) and the ‘left’ group (*p* < 0.0001). There is no significant difference between the ‘right’ group and the ‘left’ group (*p* = 0.54). These results suggest that the ‘both’ group has unique DAP values compared to the other two groups. The highest DAP values were observed in the two-sided studies, while the lowest were found in the right-sided studies. Additionally, Dunn’s post hoc tests for DAP revealed similar patterns. The ‘both’ group showed significant differences in DAP values compared to both the ‘right’ group (*p* < 0.0001) and the ‘left’ group (*p* < 0.0001). In contrast, no significant difference was found between the ‘right’ group and the ‘left’ group (*p* = 0.64). This reinforces the conclusion that the ‘both’ group exhibits distinct DAP characteristics relative to the other groups, further highlighting the differences in procedural outcomes based on the study design. The highest KERMA and DAP values were for studies that considered both sides of the patient. For left- and right-sided studies, the mean values of DAP and KERMA were lower than for bilateral studies. The lowest values were obtained for patients with only right-sided examinations. All values are shown in Table 3.

**Figure 7:**
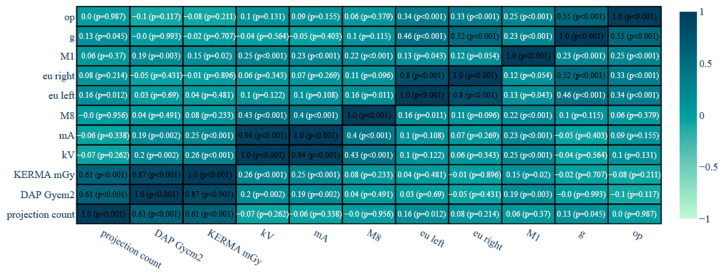
Spearman rank correlation for continuous data.

Spearman rank correlation showed a number of relationships within continuous variables (Figure 7). Among these, the relationships relevant to the subject of the study, in accordance with the theme of the study, were those between DAP and KEMA (as the most important parameters determining patient and staff exposure during interventional radiology procedures) and variables such as the number of projections, X-ray tube parameters (kV and mA) and patient skull size. Statistical analysis showed a very high correlation (rS = 0.87) between DAP (Gycm^2^) and KERMA (mGy). In addition, a high correlation was confirmed between the number of projections and DAP and KERMA (rS = 0.61). A low correlation was found between KERMA values and tube performance for mA (rS = 0.25), and kV (rS = 0.26), respectively. For the DAP value, on the other hand, no correlation was shown with mA (rS = 0.19), and a low correlation was shown with kV (r = 0.2). The value of DAP and KERM also did not depend on the thickness of the patient’s skull.

## 4. Discussion

The analysis results showed significant correlations between various examination parameters and the amount of radiation dose received. Foremost, examinations considering both sides of the patient had the highest DAP and KERMA values, while unilateral examinations (left or right side) had lower average values. Moreover, statistical correlation analysis showed a high correlation between DAP and KERMA, confirming the relevance of these two parameters in assessing a patient’s radiation exposure. 

In addition, Spearman rank correlation analysis revealed several significant correlations between continuous variables and DAP and KERMA values. Of note is the high correlation between the number of projections and radiation dose, suggesting that the number of forecasts performed significantly impacts the total radiation dose received by the patient. In addition, the tube operating parameters (kV and mA) and the size of the patient’s skull also showed significant correlations with DAP and KERMA values.

From the above, it is necessary to consider a variety of factors when planning and performing cerebral vascular DSA procedures, to minimize patient radiation exposure and ensure the safe and effective use of this diagnostic modality. Optimization of imaging protocols and consideration of individual patient characteristics can help reduce radiation exposure risks and improve the safety of patients undergoing DSA examinations.

Interestingly, the research problem we addressed has so far been addressed extremely rarely in the literature. Studies have mostly considered a selective parameter.

In contrast, a study by Ho et al. [5] stands out, containing a comprehensive analysis of radiation dose in DSA, taking into account patient age, sex, diagnoses, total examination time and the angiography technique used. The dose was described based on DAP and KERMA.

A study conducted by Chaoqun et al. [6] showed another factor influencing the increase in total DSA treatment time. This study examined the relationship between intracranial artery stenosis (IAS) pathology and DSA operational parameters. 

One should not neglect the study conducted by Li et al. [7]. The study deals primarily with radiation doses received by patients of different ages, organ-specific doses, and the risks associated with the development of cancer. The newborn’s abdomen–pelvis scan had double the effective dose of the chest scan, with a tenfold higher risk of thyroid cancer compared to the teenager. Overall, the newborn faced four times greater cancer risk.

A retrospective analysis revealed significant correlations between specific examination variables and DAP and KERMA values in interventional radiology procedures. Compared to the study by Ho et al. [5], which analyzed the impact of age, gender, and diagnosis on Ka,r and PKA values, our study focused on the number of projections, X-ray tube parameters, and the presence of embolization coils. The findings of Chaoqun et al. [6], which showed that IAS pathology prolongs DSA time without significantly affecting radiation exposure, partially align with ours, indicating no significant differences related to gender and the presence of coils. The study by Huda et al. [8] emphasized the importance of considering patient-specific factors, such as age and anatomical conditions, for a more accurate assessment of radiation exposure, which is also reflected in our results. Our study, in agreement with other studies, highlights the need for a more personalized approach to evaluating and managing radiation exposure, taking into account individual patient characteristics and specific examination conditions.

Other studies have focused attention on different DSA techniques and ways to minimize dose. Bosowska et al. [9] analyzed the differences between radiological doses in monoplane and biplane studies. Song et al. [10] suggested modifying the craniocaudal angle in DSA to reduce the dose. The radiation dose is higher at the craniocaudal angle (CC) compared to the PA angle, and the use of a copper filter significantly reduces the radiation dose.

Our study has several important limitations that may affect the interpretation of the results and generalization of conclusions. First, the retrospective nature of the study comes with limitations related to the quality and completeness of the data collected. The possibility of omitting certain important variables and the lack of uniform control over the study’s parameters can lead to bias. In addition, the study was conducted at a single medical facility, which may limit the ability to generalize the results to other centers with different protocols and equipment.

Another limitation is the relatively small and homogeneous group of patients. The lack of diversity in terms of patients’ age, gender and health status may affect the limited ability to extrapolate results to a wider population. In addition, the measurement of radiation dose was based on specific indices (DAP and KERMA), which, despite their universality, may not fully capture the totality of radiation exposure, especially in the context of local doses in specific brain areas.

To overcome these limitations, future studies should strive for prospective data collection to better control study parameters and minimize bias. The inclusion of a larger and more diverse group of patients may improve the ability to generalize results and provide more comprehensive conclusions.

Moreover, the use of advanced analytical techniques and the inclusion of new indicators for measuring radiation dose, such as organ-specific dose, may contribute to a more precise assessment of radiation exposure and its potential health effects. The introduction of multicenter comparative studies could also enable the comparison of different DSA protocols and equipment, which would provide more nuanced data and recommendations for optimizing imaging procedures.

The results of the study based on the GE Innova IGS 630 provide valuable information, but their general applicability is limited to devices of the same type. Further comparative studies with other devices and adaptation of clinical protocols and procedures to account for technological differences are necessary to ensure wider application. It is also important to educate healthcare professionals to better understand how to optimize radiation dose in different clinical settings. It is worth noting, however, that other authors, such as Ho et al. [5] or Huda et al. [8], obtained similar results.

The clinical implications of the study highlight the need for careful planning and optimization of DSA procedures to minimize patient radiation exposure. Optimization of imaging protocols and individualized adjustment of examination parameters to patient characteristics can significantly reduce the risk of radiation exposure, which is key to improving the safety and diagnostic efficacy of this modality. Taking into account variables such as the number of projections, tube operating parameters and the size of the patient’s skull would allow more precise management of radiation dose, contributing to better management of patients’ health risks.

## 5. Conclusions

The radiation dose during a DSA examination depends on factors like procedure duration, number and angle of projections, and patient characteristics such as age, gender, and existing medical conditions. This study analyzes the impact of examination parameters and patient characteristics on radiation dose in cerebral vascular DSA, using data from 251 procedures with the GE Innova IGS 630 biplane device.

Statistical analysis revealed that bilateral examinations had the highest dose area product (DAP) and air kerma (KERMA) values, while unilateral examinations had lower values. Significant correlations were found between study sides and DAP (*p* < 0.0001) and KERMA (*p* < 0.0001). Spearman’s rank correlation showed a high correlation between DAP and KERMA (rS = 0.87) and between the number of projections and radiation dose (rS = 0.61). Lamp operating parameters (kV and mA) had low correlation with DAP and KERMA values.

The findings suggest that limiting the number of projections and considering unilateral angiography when clinically justified can reduce radiation exposure. Optimizing imaging protocols and tailoring examination parameters to patient characteristics can enhance safety for both patients and healthcare professionals by minimizing radiation exposure.

## Figures and Tables

**Table 1:** Contingency table describing the population.

Sex	Coil	Side		
		L	L + R	R
F	No	17	44	22
	Yes	34	37	27
M	No	6	23	12
	Yes	15	11	3

**Table 2:** Mann–Whitney U test results for the relationship between sex, coil presence and DAP and KERMA.

Categorical Variable	Continuous Variable	U Statistics	*p* Value
coils	KERMA	7440	0.451
coils	DAP	7117	0.188
sex	KERMA	5415	0.075
sex	DAP	5344	0.055

**Table 3:** Average DAP and KERMA values depending on the number of sites tested.

Side	KERMA (mGy)	DAP (Gycm^2^)
L	90.090333	11.700239
L + R	131.510217	18.163709
R	80.048250	10.279663

## Data Availability

The original contributions presented in the study are included in the article. Further inquiries can be directed to the corresponding author.

## Definitions

DAP—dose area product (DAP) in mGycm^2^ for a specific fluoroscopic examination. It is defined as the product of the radiation dose and the surface at which the radiation is directed [11,12].

KERMA (Kinetic Energy Released per unit MAss)—the amount of kinetic energy transferred to charged particles per unit mass of matter (tissue) by ionizing radiation. It is expressed in grays (Gy) [13].

## References

[1] Singh D.K., Yadav K., Singh A.K., Sinha K., Kaif M., Kumar R., Chand V.K. (2023). Digital Subtraction Angiography of Cerebral Vessels: Basic Technique. Neurol. India.

[2] Settecase F., Rayz V.L., Hetts S.W., Cooke D.L. (2021). Chapter 6—Advanced vascular imaging techniques. Handbook of Clinical Neurology Interventional Neuroradiology.

[3] Alexander M.D., Oliff M.C., Olorunsola O.G., Brus-Ramer M., Nickoloff E.L., Meyers P.M. (2010). Patient radiation exposure during diagnostic and therapeutic interventional neuroradiology procedures. J. NeuroInterv. Surg..

[4] Wagner L.K., Eifel P.J., Geise R.A. (1994). Potential Biological Effects Following High X-ray Dose Interventional Procedures. J. Vasc. Interv. Radiol..

[5] Yi H.J., Sung J.H., Lee D.H., Kim S.W., Lee S.W. (2017). Analysis of Radiation Doses and Dose Reduction Strategies During Cerebral Digital Subtraction Angiography. World Neurosurg..

[6] Guo C., Shi X., Ding X., Zhou Z. (2018). Analysis of Radiation Effects in Digital Subtraction Angiography of Intracranial Artery Stenosis. World Neurosurg..

[7] Li X., Samei E., Segars P.W., Sturgeon G.M., Colsher J.G., Toncheva G., Yoshizumi T.T., Frush D.P. (2011). Patient-specific radiation dose and cancer risk estimation in CT: Part II. Application to patients. Med. Phys..

[8] Huda W., Lieberman K.A., Chang J., Roskopf M.L. (2004). Patient size and X-ray technique factors in head computed tomography examinations. I. Radiation doses. Med. Phys..

[9] Bosowska J., Modlińska S., Pękala T., Szydło F., Cebula M. (2022). Impact of monoplane to biplane angiography upgrade on diagnostic angiography procedures: A retrospective cross-sectional study. Phys. Medica.

[10] Song Y., Kim Y., Han S., Kim T.I., Choi J.H., Maeng J.Y., Choi Y., Lee D.H. (2019). Estimated radiation dose according to the craniocaudal angle in cerebral digital subtraction angiography: Patient and phantom study. J. Neuroradiol..

[11] Vañó E., Miller D.L., Martin C.J., Rehani M.M., Kang K., Rosenstein M., Ortiz-López P., Mattsson S., Padovani R., Rogers A. (2017). ICRP Publication 135: Diagnostic Reference Levels in Medical Imaging. Ann. ICRP.

[12] Vano E., Järvinen H., Kosunen A., Bly R., Malone J., Dowling A., Larkin A., Padovani R., Bosmans H., Dragusin O. (2008). Patient dose in interventional radiology: A European survey. Radiat. Prot. Dosim..

[13] Attix F.H. (2004). Introduction to Radiological Physics and Radiation Dosimetry.

